# Motor development in the first year of life predicts impairments in cognition and language at 3 years old in a Brazilian preterm cohort of low-income families

**DOI:** 10.3389/fnins.2022.1034616

**Published:** 2022-10-12

**Authors:** Carolina Panceri, Rita C. Silveira, Renato Soibelmann Procianoy, Nadia Cristina Valentini

**Affiliations:** ^1^Department of Physical Education, Physiotherapy and Dance, Universidade Federal do Rio Grande do Sul (UFRGS), Porto Alegre, RS, Brazil; ^2^Department of Physical Education and Occupational Therapy, Hospital de Clínicas de Porto Alegre, Porto Alegre, RS, Brazil; ^3^Department of Pediatrics and Neonatology, Universidade Federal do Rio Grande do Sul (UFRGS), Porto Alegre, RS, Brazil; ^4^Neonatal Unit, Hospital de Clínicas de Porto Alegre, Porto Alegre, RS, Brazil

**Keywords:** child development, preterm, neurodevelopmental disorders, motor skills, cognition, language development, low-income population

## Abstract

**Purpose:**

Early identification of impairments is crucial to providing better care for preterm children, especially those from low-income families. The early motor assessment is the first step in monitoring their neurodevelopment. This study investigates if motor development in the first year of life predicts impairments in cognition and language at 3-year-old in a Brazilian preterm cohort.

**Materials and methods:**

Data were collected in a follow-up clinic for high-risk infants. The Bayley Scales were used to assess children at 4, 8, 12, and 36 months of age, considering composite scores. Cognitive and language impairments were considered if scores were ≤85. Children (*N* = 70) were assessed at 4 and 36 months, 79 were assessed at 8 and 36 months, and 80 were assessed at 12 and 36 months. Logistic regressions were used to analyze the predictability of cognitive and language impairments, and receiver-operating characteristics (ROC) curves were used to analyze the sensibility and specificity of motor assessment and cognitive and language impairments.

**Results:**

Poor motor scores at 8 and 12 months increased the chances of cognitive and language impairment at 3-year-old. The chance of cognitive impairment at 3-year-old increases by 6–7% for each point that the motor composite score decreases, and the chance of language impairment at 3-year-old increases by 4–5% for each point that the motor composite score decreases. No-significant results were found at 4-months. Adequate sensibility and specificity were found for language impairments considering 12 months scores and for cognitive impairments as soon as 8 months scores.

**Conclusion:**

Monitoring preterm motor development in the first year of life helps to identify preterm children at risk for impairment in other developmental domains. Since preterm children from low-income families tend to demonstrate poorer neurodevelopment outcomes, these children need early assessment and referral to intervention to prevent school failures and support from public policies.

## Introduction

Children born preterm have higher neurodevelopment impairments rates than their term peers (Beauregard et al., 2018; Sanchez et al., 2019; Valentini et al., 2019; Zuccarini et al., 2020; Sandoval et al., 2021). They have a high risk for delays across multiple domains like motor, cognitive, language, or social development, even without major cerebral damage (Aylward, 2005). Pre-school-age preterm children exhibit deficits in executive function (inhibitory control, cognitive flexibility, working memory, and planning/executive functioning) (Sandoval et al., 2021) and receptive and expressive language (Sanchez et al., 2019). The early identification of impairments allows intervention as early as possible, preferably before school age, to prevent academic difficulties and failures. However, although motor differences can be noticeable at 2-months of age or even earlier for preterm children (Aylward, 2005; Sampaio et al., 2015), cognitive and language deficits do not manifest until older ages–most at a pre-school or older age (Sanchez et al., 2019; Zuccarini et al., 2020). However, even before cognitive and language impairments are noticeable, these children could benefit from early intervention and perhaps minimize the difficulties in the child’s life and academic performance. The earlier the intervention, the more meaningful the benefits are since the baby’s learning capacity is optimized by neuroplasticity–the ability of the central nervous system (CNS) to modify its structural and functional organization in response to the action of environmental stimuli.

Socioeconomic and biological risk factors have been extensively examined in their role in predicting the neurodevelopment outcome for preterm children (Saccani et al., 2013; Velikos et al., 2015; Chiquetti et al., 2018; Silveira et al., 2018; Panceri et al., 2020). Overall, the diversity of the results highlights the need to examine this multifactorial phenomenon further. Our contribution is to investigate the neuromotor responses in the first year of life at different ages to predict subsequent development in cognitive and language domains. Although some studies addressed these questions (Oudgenoeg-Paz et al., 2017; Sanchez et al., 2019), none were conducted with low-income children for LMIC (Low- and Moderate-Income Countries). Movements are one of the primary developmental responses in early life. After birth, the primitive reflexes subserve essential functions that facilitate the infants’ survival, such as feeding and protection, whereas postural reflexes involve responses to changes in orientation relative to the environment (Clark, 2005). Later, voluntary movements facilitated infants’ communication through gestures, exploration of objects, and action upon exploring the environment through locomotion. Therefore, through voluntary movements, children become independent. Consequently, the early motor assessment may be the first step to monitoring preterm’ neurodevelopment and providing adequate interventions, even for other domains besides motor.

Multiple development domain interactions are observed in the first years of life (Nip et al., 2011; Walle and Campos, 2014). Children develop cognitive and language skills through continuous interaction with their environment and the people around them. The physical exploration of the environment, which requires locomotion and manipulation skills, allows learning new possibilities within the environment, objects, and interaction with others (Nip et al., 2011; Walle and Campos, 2014; Oudgenoeg-Paz et al., 2017). Thus, especially in the child’s first year of life, motor, cognitive, and language development seem to occur interdependently (Campos et al., 2012). However, there is still scarce empirical evidence linking early motor development with later cognitive and language outcomes in preterm children from Low- and Moderate-Income Countries (LMIC). This information is crucial to provide the best intervention that can lead to better cognition and language outcomes and support children living in poverty to achieve their full potential.

Thus, this study aimed to investigate if motor development in the first year of life predicts impairments in cognition and language at 3-year-old in a Brazilian preterm cohort. We hypothesize that motor performance is the first sign to detect impairments in other developmental domains and that it is possible to detect that in the first year of life.

## Materials and methods

### Context and participants

This study is part of a cohort study in a Brazilian public hospital, where most patients come from low-income families with low formal education. All inborn preterm infants with less than 32 weeks of gestation and/or birth weight less than 1,500 g are referred to the Neonatology Outpatient Clinic and included in the present study. They were enrolled in monthly follow-up multidisciplinary appointments until 6 months of corrected age, bi-monthly from 7 to 12 months of corrected age, every 3 months from 8 to 24 months of corrected age, and once a year until 5-year-old, following the hospital practice routine. The children in this study were followed from March, 2017 to March, 2021. Preterm with congenital malformations and genetic syndromes diagnosed by the neonatologist were excluded. All parents or legal guardians signed the informed consent, and the institutional ethics committee approved the study.

### Assessments

Children’s biomedical data regarding the hospital stay and follow-up were prospectively collected (i.e., gestational age, birth weight and length, head circumference, APGAR, mechanical ventilation, periventricular hemorrhage, and periventricular leukomalacia), and the parents completed a survey related to socioeconomic data.

The Bayley Scales of Infant and Toddler Development third edition (BSITD-III) were used to assess children’s cognitive, language (receptive and expressive), and motor (fine and gross) development; corrected age for the first year of life (4, 8, and 12 months) and chronological age at 36 months were used. The BSID-III is standardized and widely recognized in the literature as a golden standard tool to assess child neurodevelopment. The BSID-III scores the child’s performance according to their age. The raw score is provided by the sum of all items the child received credit for, plus the sum of items from previous ages. A composite score, derived from raw scores and considering the child’s age, was used in the present study. The BSITD-III composite scores have a standardized mean of 100 with standard deviations (SDs) of 15 points. Following the recommended guidelines, impairment categorization was detected if the composite scores were less than 85 (−1SD) (Bayley, 2005).

### Controlling for confounding factors

We assessed the home environment and maternal practices using three instruments to control for confounding factors. The Knowledge of Infant Development Inventory–KIDI adapted for Brazilian children, was used to assess parental knowledge regarding infant development; it contains, for this age group, 20 items regarding the age at which infants develop specific skills. The total score is obtained by the ratio between the correct answers and the total item (1 is the maximum score) (MacPhee, 1981; Ribas et al, 2000). The Interaction Rating Scale (IRS) was used at 12 months to assess the mother/child dyads. The IRS is an observational tool focused on a child’s social skills, the caregiver’s parenting skills, and the caregiver/child interactions (Amne, 2009). The scale has 70 dichotomous items (1 = yes, 0 = no), and the sum of all items gives the overall observed behavior score. The validation process for the Brazilian population is in progress by the present research group. Affordance in the Home Environment for Motor Development–Infant Scale–AHEMD-IS, to evaluate development opportunities available at home regarding physical space, outside and inside daily activities, and play materials; total score and categorization are provided (less than adequate: 0–18; moderate adequate: 19–23; adequate: 24–27; excellent: 28–49) (Bartlett et al., 2008; Caçola et al., 2011).

### Procedures

**FIGURE 1:**
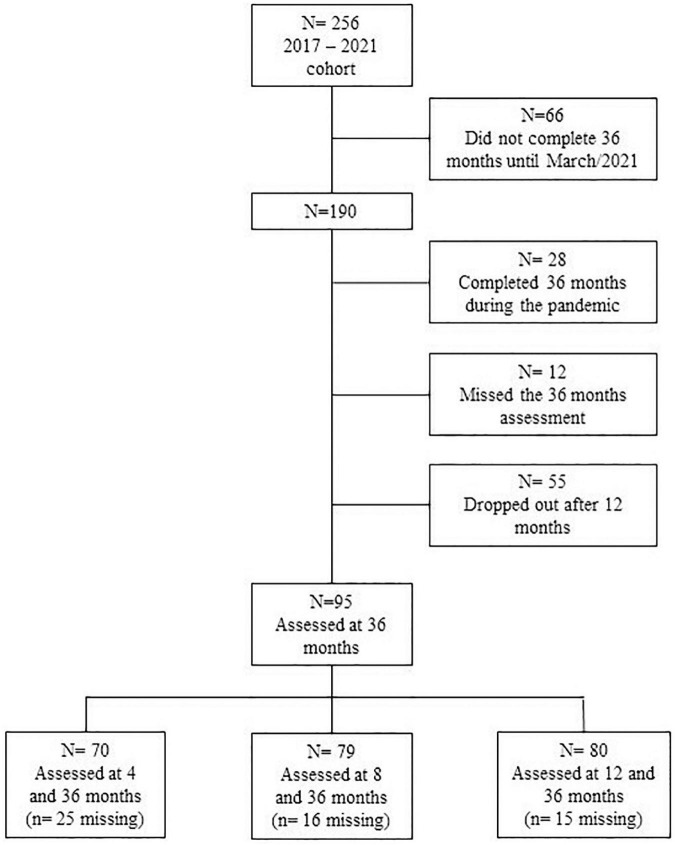
Participants enrollment in the study.

The public hospital ethics committee approved the study (process *n*° 2019-0321); the study was conducted in the Neonatology Outpatient Clinic following the norms established by Resolution 466/12 of the National Health Council. The BSITD-III assessment is part of the preterm follow-up protocol in the outpatient clinic established by the researchers of the present study. The BSITD-III assessments for the present study were conducted at pre-established visits at 4, 8, and 12 months of corrected age and 36 months of chronological age. Some children did not attend some appointments and therefore did not have data at all time points. Further, some children did not complete 36 months until March, 2021, and some completed during the COVID-19 pandemic, so it was impossible to carry out the assessment. Consequently, our study included 70 children assessed at 4 and 36 months, 79 at 8 and 36 months, and 80 at 12 and 36 months. There was missing at random in the present sample; the participant’s enrollment across the study and missing data are described in Figure 1.

The parents or legal guardians were present at the children’s assessment sessions. Two previously trained professionals with more than 5 years of experience using the BSITD-III conducted all assessments together. They scored and analyzed the children’s performance independently and then compared the results of 50% of the sample; inter-rater reliability was high (ICC >85).

### Data analyses

The sample size calculation was performed based on the prevalence of delays throughout the follow-up using the GPower 3.1. The analysis was run considering the lower expected prevalence of 20% (Valentini et al., 2021), a minimum significance level of 5%, and power of 80%–the minimum total of 50 children per group/age was obtained.

Means, standard deviations, and frequencies were estimated. The student’s *t*-test was used to compare quantitative variables, and the Chi-square was used for testing relationships between categorical variables. Correlations were conducted considering coefficients below 0.30 weak, those between 0.30 and 0.70 moderate, and coefficients above 0.70 strong. BSITD-III motor composite scores at 4, 8, and 12 months were analyzed using logistic regression as predictors of cognitive and language impairment at 36 months. Impairments were classified as BSITD-III composite scores less than 85. Sensitivity, specificity, and positive and negative predictive values with 95% CI were used to assess the BSITD-III motor composite scores at 4, 8, and 12 months as a diagnostic tool for predicting cognitive and language impairment at 36 months. The trade-off between the sensitivity and specificity of a range of cut-off points on the BSITD-III was examined using receiver-operating characteristics (ROC) curves.

## Results

### Controlling for confounding factors

Since we investigated the potential prediction of motor development on cognition and language, environment confounding factors that could influence child development were controlled within our research design to ensure results validity. No significant differences were found between groups (cognitive impaired or typical; and language impaired or typical) for the maternal care routine, mother knowledge regarding child development, and home opportunities for development. Table 1 presents the controlled confounding factors.

**TABLE 1:** Maternal practice and knowledge, and home opportunities.

Assessment tools	Cognitive groups			Language groups		
		
	Impaired *N* = 36	Typical *N* = 59	*p*	Impaired *N* = 36	Typical *N* = 59	*p*
KIDI–Parents Knowledge	0.59 (0.09)	0.61 (0.09)	0.430	0.60 (0.09)	0.60 (0.09)	0.842
IRS–Interaction Rating Scale	49.75 (16.12)	55.98 (17.4)	0.374	46.35 (20.98)	58.09 (13.62)	0.130
AHEM–Home Affordances for Development	51.80 (12.23)	54.38 (15.64)	0.093	52.65 (12.47)	53.36 (14.75)	0.082

Values are mean (standard deviation).

### Neurodevelopment outcomes

The mean age at the 4 months assessment for the 95 participants in this analysis was 4.57 (SD 0.57); at 8 months, it was 8.41 (SD 1.01); at 12 months, it was 12.84 (SD 1.13); and, at 36 months it was 33.20 (SD 5.47). Table 2 provides biomedical and environmental characteristics for the participants assessed at 36 months (the children who remain in the study), the group that missed the 36 months assessment and dropped out from the study, and the statistical comparisons across groups. Results demonstrate significant differences between groups in clinical characteristics (gestational age, birth weight, length, head circumference, days of neonatal intensive care, and days of mechanical ventilation), the number of children assessed at each age, and BSITD-III motor composite scores at each age. Children who lost the follow-up assessment at 36 months had better clinical outcomes and higher motor scores at 12 months and attended fewer assessment sections in the first year of life.

**TABLE 2:** Biomedical and environmental characteristics of the cohort for children follow up at 36-months and children that dropped out.

Characteristics	Children followed-up to 36 months (*n* = 95)	Children dropped out (*n* = 95)	*p*
Gender (%)			0.335
Boys	53 (55.2)	48 (51.1)	
Girls	43 (44.8)	46 (48.9)	
Gestational age (weeks)	29.03 (2.52)	30.50 (2.10)	≤0.001*
Birth weight (grams)	1,121.40 (329.99)	1,362.73 (377.74)	≤0.001*
Birth length (centimeters)	36.63 (4.03)	39.18 (3.42)	≤0.001*
Head circumference	26.21 (2.66)	27.54 (2.38)	0.001*
Small for gestational age (%)	23 (24)	27 (28.7)	0.353
APGAR 5th min	7.81 (1.48)	7.9 (1.79)	0.703
Neonatal intensive care unit stay (days)	76.64 (39.30)	53.22 (31.50)	≤0.001*
Mechanical ventilation (days)	9.30 (18.98)	3.23 (7.89)	0.006*
Periventricular hemorrhage (%)			0.373
Grade 0	63 (65.7)	71 (75.5)	
Grade 1 and 2	30 (41.2)	18 (19.2)	
Grade 3 and 4	3 (3.1)	5 (5.3)	
Periventricular leukomalacia (%)			0.566
Yes	5 (5.2)	5 (5.3)	
No	91 (94.8)	89 (94.7)	
Mother age at infant’s birth	27.46 (6.40)	28.69 (7.03)	0.211
Father age at infant’s birth	30.31 (6.99)	32.89 (10.79)	0.075
Family income (R$)	2,215.29 (1,554.80)	2,234.75 (1,313.66)	0.936
Mother formal education (%)			0.214
Less than high school	38 (39.6)	40 (43.5)	
High school	46 (47.9)	41 (43.8)	
College	12 (12.5)	12 (12.7)	
Father formal education (%)			0.184
Less than high school	47 (49.0)	47 (49.9)	
Complete high school	45 (46.8)	39 (41.6)	
College	4 (4.2)	8 (8.5)	
Assessed at 4 months (%)	70 (72.91)	48 (50.52)	0.003*
BSITD-III motor composite score at 4 months	(16.81)	94.40 (17.77)	0.738
Assessed at 8 months (%)	79 (82.29)	49 (51.57)	≤0.001*
BSITD-III motor composite score at 8 months	87.06 (17.19)	89.80 (16.34)	0.374
Assessed at 12 months (%)	80 (83.33)	46 (47.91)	≤0.001*
BSITD-III motor composite score at 12 months	87.21 (20.32)	95.49 (19.81)	0.028*

Unless otherwise noted, values are mean (standard deviation).

*p ≤ 0.05.

In the follow-up group, at 4 months, 21 (30%) of the 70 children demonstrated motor impairments; at 8 months, 35 (44.3%) of the 79 children; and, at 12 months, 35 (43.8%) of the 80 children. At 36 months, 36 (37.5%) of the 95 children showed cognitive impairments and 36 (37.5%) language impairments. Overall, 45 (46.8%) children had cognitive or language impairments at 36 months.

Correlation analysis showed BSITD-III motor composite scores at 8 months were significant, moderate, and positively related to cognitive and language composite scores at 36 months. Moderate, positive, and significant correlations between the motor score at 12 months and cognitive and language scores at 36 months were observed. The motor scores at 4 months were non-significant correlated with cognitive (*p* = 0.767) or language (*p* = 0.963) scores at 36 months. Table 3 provides correlation values.

**TABLE 3:** Correlations of the motor outcomes at 4, 8, and 12-months with cognitive and language outcomes at 36 months.

	BSITD-III cognitive composite score at 36 months	BSITD-III language composite score at 36 months
BSITD-III motor composite score at 4 months	0.036	0.006
BSITD-III motor composite score at 8 months	0.531**	0.471**
BSITD-III motor composite score at 12 months	0.660**	0.520**

Pearson coefficient correlation values.

**p ≤ 0.001.

Motor performance at 8 months was a significant predictor for cognitive and language impairments at 36 months. The chance of cognitive impairment at 3-year-old increases by 6.4% (95% CI: 3.0 – 10.7, *p* = 0.001) for each point that the motor composite score decreases; and the chance of language impairment at 3-year-old increases by 5.2% (95% CI: 2.1–9.0, *p* = 0.002) for each point that the motor composite score decreases. At 12 months, motor performance was also a significant predictor of cognitive and language impairment at 36 months. The chance of cognitive impairment at 3-year-old increases by 7.2% (95% CI: 3.8–11.7, *p* ≤ 0.001) for each point that the motor composite score decreases; and the chance of language impairment at 3-year-old increases by 4.2% (IC 95%: 1.2–7.1, *p* = 0.003) for each point that the motor composite score decreases. At 4 months, no significant associations were detected for cognition (OR 2.2, 95% CI: −1–5.7, *p* = 0.178) or language (OR 0.9, 95% CI: −1.9–4.0, *p* = 0.525).

**FIGURE 2:**
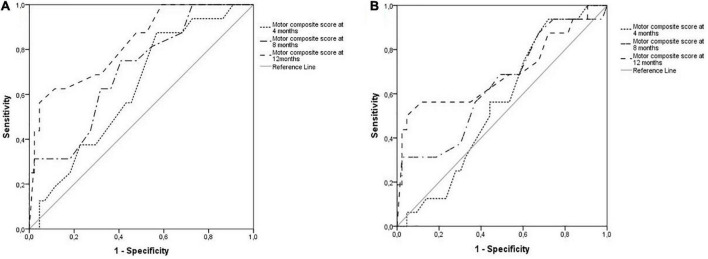
ROC curves for cognition **(A)** and language **(B)** impairments.

The sensibility and specificity of the motor assessment at 4 months were non-significant for cognitive (*p* = 0.137) or language (*p* = 0.534) impairments at 36 months. The motor assessment at 8 months showed no significant sensibility and specificity for language impairments (*p* = 0.100). Cognitive impairments were possible to identify at 8 months; the area under the curve (AUC) was 0.71 (95% CI 0.56–0.85; *p* = 0.014), the best cutoff point of the BSITD-III motor composite score was 89.5 with 75% sensibility and 59.1% specificity. At 12 months, cognitive and motor impairments were possible to identify; the AUC was 0.82 (95% CI 0.71–0.94; *p* ≤ 0.001) for cognitive and 0.69 (95% CI 0.52–0.87; *p* = 0.022) for language impairments. The best cutoff point for BSITD-III motor composite score was 77.50 for cognitive (53.3% sensibility and 95.5% specificity) and language (50% sensibility and 95.3% specificity) impairments. Figure 2 presents the curves for cognitive (A) and language (B) impairments.

## Discussion

The present study investigated whether motor development in the first year of life predicts impairments in cognition and language at 3-year-old in a Brazilian preterm cohort of children from low-income families. The results indicate that poor motor scores assessed with the BSITD-III at 8 and 12 months of corrected age increase the chances of cognitive and language impairment at 3-year-old. There were no significant results at the 4-month of age assessments. The ROC curves showed that in the 12 months of life, the motor assessment has an adequate sensibility and specificity to identify language impairments at 36 months, whereas cognitive impairments were possible to identify as soon as 8 months.

Our results are aligned with previous research findings. A study investigating exploratory object behaviors and their relation to later language and cognitive development with preterm infants found that oral and manual exploration of objects at 6 months were related to language and cognitive development at 24 months (Zuccarini et al., 2017). The authors recorded 5 min of play and coded exploratory behaviors at 6 months. At 2-year-old, children were assessed by the Griffiths Mental Developmental Scales 0–2 (Zuccarini et al., 2017). Another study also reported strong associations between reaching at 8 months and cognition and language development at 30 months for preterm children (Kaul et al., 2019). The children’s movements were recorded by a 3D capture system at 8 months and assessed later at 2.5 years of age with BSITD-III. The authors suggested that reaching could be an early executive function marker and that early in life, motor control and executive function could share a common base (Kaul et al., 2019). Our results support these contentions.

Furthermore, studies that used standardized assessment tools reported similar results at different ages. Gross motor skills assessed at 6 months by Griffiths Mental Developmental Scales were associated with cognitive skills at 12 months with the same instrument (Zuccarini et al., 2020). The Prechtl General Movement Assessment (GMA) also was associated with cognitive outcomes assessed with different tools from 2 to 11 years old (Einspieler et al., 2016). Besides, a review focused on evidence for a link between motor development and later cognitive skills in preterm children reported a relatively consistent relationship between the level of early motor development, quality of postural control or general movements, and later cognitive skills (Oudgenoeg-Paz et al., 2017), no data on language was examined. Although, studies involving language report results that corroborate our findings. The results on the Test of Infant Motor Performance were significantly associated with later language and cognitive development assessed by BSITD-II at 2-year-old (Peyton and Einspieler, 2018).

A plausible explanation for these results is the intrinsic nature of motor exploration. Motor acquisitions allow children to practice skills relevant to cognition and language acquisition (Iverson, 2010). The achievement of motor milestones, such as reaching, sitting without support, crawling, and walking, radically alters the child’s relationship with the objects and people in their environment. Infants who have started reaching find a whole new set of opportunities for manipulating and exploring objects. Infants who sit unsupported can freely rotate their heads and trunk, improving their visual observation of the world around them from different angles. Besides, they have arms and are hands-free to explore objects around them. When they acquire the ability to move, first crawling and then walking, the possibilities of social interaction increase and change the information children receive from their environment (Iverson, 2010; Oudgenoeg-Paz et al., 2016). Thus, the child needs to interact with others to advance cognitive and language acquisition (Iverson, 2010; Oudgenoeg-Paz et al., 2016; Borchers et al., 2019).

The cascading effects of early motor development on other developmental domains in preterm children have been highlighted in several studies (Zuccarini et al., 2020). Developmental changes in one domain can have cascading effects on others, even seemingly unrelated ones–they can be direct or indirect as well as multidirectional (Iverson, 2021). However, none of the studies addressed preterm children from LMIC, so here, we advance the previous knowledge by providing strong evidence of this relationship between motor with later cognitive and language development for preterm children from low-income families and LMIC.

Besides, unlike other studies (Fjørtoft et al., 2013; Einspieler et al., 2016; Peyton and Einspieler, 2018), we did not find significant results at the early age of 4 months. At this age, most of the infants in our study could not reach when sitting with support, and none of them could sit unsupported (milestones often associated with late language and cognitive development); this is a possible explanation for our findings. Previous studies have provided evidence of those associations, even for younger children. For example, spontaneous movement assessed at 11–16 weeks post-term age was positively associated with later intelligence at 7 and 11 years in children born less than 33 weeks of gestation (Borchers et al., 2019). Further, neuromotor development assessed by Touwen’s Neurodevelopmental Examination at 9 and 15 weeks old was associated with an increased risk of non-verbal cognitive delays at 2.5 years old (van Batenburg-Eddes et al., 2013). However, using BSITD-III in our study, we could predict later development from 8 months of age for extremely preterm children, extending the actual knowledge in this field with the most widely used tool for early development assessment. However, as detected in a previous study (Anderson and Burnett, 2017), the BSITD-III may be less sensitive at a very young age (4 months or less) to predict late functioning. Therefore, further studies are still necessary to examine this issue, perhaps using the 4th edition of Bayley Scales.

A particular strength of this study is the long-term follow-up of neurodevelopmental outcomes for low-income preterm children living in an LMIC; to our knowledge, this is the first study in Latin America. Investigating neurodevelopmental outcomes in Brazil and other low and moderate-income countries is essential to provide better care for children. Since preterm children from low-income families tend to demonstrate poorer neurodevelopment outcomes, these children need early assessment and referral to intervention to prevent school failures and support from public policies.

Although essential to child development, studies with similar designs are still scarce in LMIC mainly due to cost, the extended time required to complete longitudinal assessments, and the high levels of participants’ drop out due to family mobility from cities and jobs. Future research should consider different strategies to maintain low-income families’ engagement in longitudinal studies. A limitation of the present study, 95 children, dropped out. However, it is vital to acknowledge that those 95 children had a higher mean gestational age and birth weight than the ones that continued the study over the years. As the aim was to investigate if motor development in the first year of life predicts impairments in cognition and language at 3-year-old, we had similar performance in the more vulnerable population. The risk of major disability increases with decreasing gestational age, lower birth weight, presence of periventricular Leukomalacia, mechanical ventilation, and extended hospital stay (Costeloe et al., 2000; Ream and Lehwald, 2018). These factors are known associated factors with neurodevelopment impairments among children born preterm. Brain growth and development inside the uterus are interrupted by preterm birth; infants born with immature brains are more vulnerable to brain damage due to immaturity and possible infections and inflammations, leading to difficulties or neurodevelopmental disorders.

Another limitation that can be mentioned is the use of only one assessment tool. Besides BSITD-III being considered the golden child development assessment standard, other scales are frequently used for research or clinical practice, such as proxy reports that could provide further information regarding children’s difficulties at home. Our long-term follow-up was up to children’s 3-year-old – before school age. Our recommendation for future studies is to follow up with these children until elementary school and assess children with specific tools for executive function (i.e., inhibitory control, working memory, processing speed, cognitive flexibility) and vocabulary acquisition and verbal comprehension, all essential prerequisites for school success. Moreover, another limitation is the lack of analysis by prematurity groups (i.e., extremely, very, moderate, late), due to sample size. Extremely preterm children (<28 weeks) may demonstrate more neurodevelopmental impairments than very or moderate preterm children. Future studies with a larger sample size should analyze the outcome according to the prematurity group.

This study focused on the association and predictability of motor development in the first year of life and later cognitive and language outcomes in preterm children from an LMIC. In sum, our findings highlighted that motor scores at 8 months predicted cognitive development at 3-year-old and motor scores at 12 months predicted language and cognition development at 3-year-old. This study highlights the importance of clinicians and researchers assessing motor milestones in the preterm first year of life to detect early motor impairment and prevent adverse outcomes in other developmental domains. Motor assessments play an essential role in identifying severe or subtle impairments. As sooner these impairments are identified, the sooner the child can be referred to physical, occupational, or speech therapy interventions. Since poor cognitive and language skills are related to low academic performance, it is critical to assess preterm children early to support family and professional strategies to improve their function before elementary school. Given the limited evidence in this field, especially in low-income countries, more research is needed to draw robust evidence regarding this issue.

## Data availability statement

The original contributions presented in this study are included in the article/supplementary material, further inquiries can be directed to the corresponding author.

## Ethics statement

The studies involving human participants were reviewed and approved by the Comitê de Ética em Pesquisa do Hospital de Clínicas de Porto Alegre. Written informed consent to participate in this study was provided by the participants’ legal guardian/next of kin.

## Author contributions

CP contributed to study design, data acquisition and interpretation, statistical analyses, and manuscript preparation. NV contributed to study design, interpretation, statistical analyses, and manuscript preparation. RS and RP contributed equally to manuscript revision and provided significant oversight on the intellectual content. All authors critically reviewed the manuscript and approved the submitted version for publication.
